# Microfluidic Preparation of pH-Responsive Microsphere Fibers and Their Controlled Drug Release Properties

**DOI:** 10.3390/molecules29010193

**Published:** 2023-12-28

**Authors:** Ning Wang, Yixuan Wei, Yanrong Hu, Xiaoting Sun, Xiaohong Wang

**Affiliations:** 1 Center of 3D Printing & Organ Manufacturing, School of Intelligent Medicine, China Medical University, Shenyang 110122, China; wangning@cmu.edu.cn; 2 Department of Chemistry, School of Forensic Medicine, China Medical University, Shenyang 110122, China; 3 Teaching Center for Basic Medical Experiment, China Medical University, Shenyang 110122, China; weiyixuan07@126.com; 4 Department of Biological Physics, School of Intelligent Medicine, China Medical University, Shenyang 110122, China; 18262637190@163.com

**Keywords:** pH response, alginate, drug delivery, dressing, microfluidics, microsphere fiber

## Abstract

In this study, a capillary microfluidic device was constructed, and sodium alginate solution and a pH-sensitive hydrophobic polymer (*p*(BMA-*co*-DAMA-*co*-MMA)) solution were introduced into the device for the preparation of hydrogel fibers loaded with polymer microspheres. The structure of the microsphere fiber, including the size and spacing of the microspheres, could be controlled by flow rate, and the microspheres were able to degrade and release cargo responding to acidic pH conditions. By modification with carboxymethylcellulose (CMC), alginate hydrogel exhibited enhanced pH sensitivity (shrunk in acidic while swollen in basic condition). This led to an impact on the diffusion rate of the molecules released from the inner microspheres. The microsphere fiber showed dramatic and negligible degradation and drug release in tumor cell (i.e., A431 and A549 cells) and normal cell environments, respectively. These results indicated that the microsphere fiber prepared in this study showed selective drug release in acidic environments, such as tumor and inflammation sites, which could be applied as a smart surgical dressing with normal tissue protective properties.

## 1. Introduction

Sodium alginate hydrogels are employed in a wide range of applications [1,2,3], especially in biomedical fields such as surgical dressings [4], drug delivery [5] and tissue engineering [6] due to their good biocompatibility, adjustable physicochemical properties, excellent gel formation and water holding properties. Alginate fibrous materials are promising candidates for tissue repair in the biomedical field because of their high surface area, ease of preparation and handling, and their capability to maintain mechanical strength in the wetted state [7,8]. Based on these merits, alginate fibers have great potential for application in the development of novel medical dressings [9,10,11]. For example, a commercial dressing named Kaltostat alginate comes into contact with body fluids with an alginate gel-fiber matrix that keeps the wound environment moist and facilitates hemostasis and helps to control minor bleeding [12,13]. Gel fibers can also be used to mimic the extracellular matrix to enhance the proliferation of epithelial cells, and the fibrous network can promote hemostasis in injured tissues, enhance fluid absorption and promote drug delivery, cellular respiration and gas permeation in the skin [14,15,16]. However, dressings made from pure alginate hydrogel fibers are incapable to produce therapeutic effects such as preventing bacterial infection and promoting tissue regeneration, especially in chronic wound healing situations [8,17]. Loading with therapeutic factors or other modifiers can improve cell adhesion, antimicrobial activity, blood coagulation and hemostasis in gel dressings [8,18], thus the development of alginate hydrogel fibers loaded with active or therapeutic components is of practical research interest.

It has been well documented that alginate hydrogels can serve as carriers for drugs and other bioactive molecules such as proteins [19,20,21]. Alginate hydrogels can also be used as tissue engineering components for the efficient encapsulation of active substances such as cells [22]. In alginate hydrogel drug delivery systems, drug release relies primarily on diffusion from the hydrogel network, lysis, chemical mediation and environmental stimulation [23,24]. However, substances with low molecular weight, such as drugs, vitamins and sugars, the molecular particle sizes of which are usually smaller than the pore size of alginate hydrogels, can diffuse freely in the network [25], which limits the loading efficiency and release control of small molecule drugs in alginate hydrogels. Therefore, it is crucial to construct alginate hydrogel materials that can provide efficient loading and release control of small molecules.

Dufresne et al. proposed a double layer alginate membrane with two antibiotic drugs loaded in different layers. The inner layer had cellulose nanocrystals in the inner layer, which enabled prolonged drug release while the outer layer of neat alginate gave rise to a rapid release. The alginate hydrogel was capable of realizing complex drug release kinetics, leading to synergistic effects in trauma healing dressing applications [26]. More recent work has also created a dual antibiotic-phage delivery system based on alginate hydrogel for the treatment of fracture-related infections [27]. However, the drug loading efficiency and controlled release performance of alginate hydrogels should be effectively improved by the composition of biomaterials.

In this study, we constructed a composite alginate hydrogel fiber encapsulated with drug-loaded microspheres based on a coaxial capillary microfluidic device to investigate its potential application as a drug-delivery dressing. The biocompatible polymers polylactic-*co*-glycolic acid (PLGA) and poly(butyl methacrylate-*co*-(2-dimethylaminoethyl) methacrylate-*co*-methyl methacrylate) (*p*(BMA-*co*-DAMA-*co*-MMA)) were used as a matrix for drug-loaded microspheres for efficient loading of small molecule drugs, and the pH-sensitive properties of *p*(BMA-*co*-DAMA-*co*-MMA) and alginate hydrogels doped with carboxymethyl cellulose were exploited to achieve pH-responsive release control of the loaded drugs. This kind of composite fiber is expected to realize the automatic adjustment of drug release rate according to the pH change at the drug delivery site and to build a kind of medical dressing with an intelligent drug release function.

## 2. Results and Discussion

### 2.1. O/W (Oil-in-Water Emulsions) Droplet Formation

**Figure 1:**
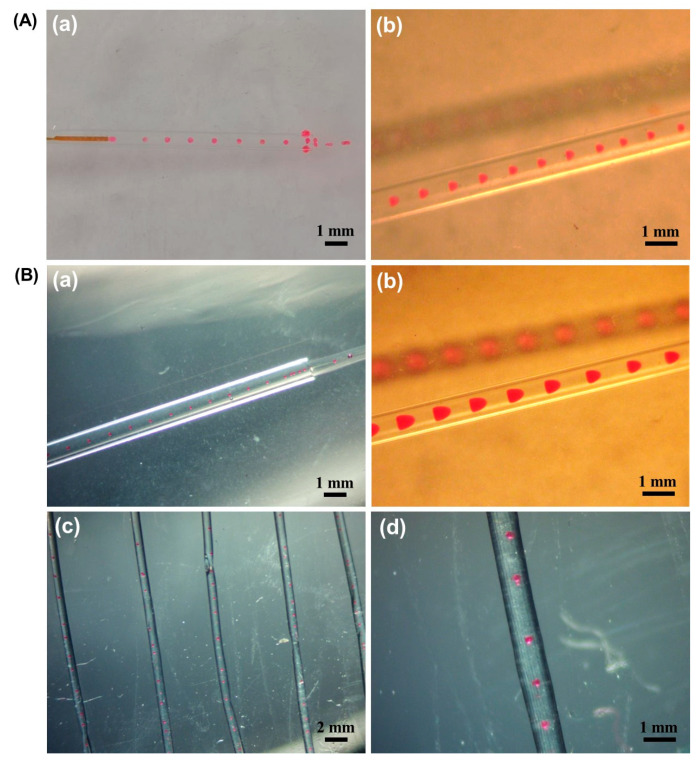
(**A**) Formation of O/W droplet (**a**) and the droplet in the capillaries (**b**). (**B**) Formation of PLGA solution droplet (**a**,**b**) and PLGA microspheres@calcium alginate fiber (**c**,**d**).

The solvent evaporation method was used in this study to prepare polymeric drug-loaded microspheres in alginate fibers. Hydrophobic polymers such as PLGA and *p*(BMA-*co*-DAMA-*co*-MMA) were proposed as microsphere materials; therefore, the formation of O/W droplets was first confirmed in the capillary microfluidic system. The solvent dimethyl carbonate (DMC) containing Nile Red dye (used as the dispersed phase, oil phase) and sodium alginate solution (used as the continuous phase, aqueous phase) were injected into the inner and outer capillaries, respectively. As shown in Figure 1A, it could be observed in the glass capillary that the oil phase was sheared by the sodium alginate solution at the exit of the quartz capillary and droplets were continuously generated after adjusting the flow rate. The droplet size and spacing are uniform, which indicates that the coaxial capillary device can be used directly for O/W droplet generation without the need for channel wall modification.

### 2.2. Microsphere Fiber Formation

In order to prepare alginate fibers encapsulated with drug-laden microspheres, the study began with PLGA as the microsphere material, which was dissolved in DMC to act as the dispersed phase. As shown in Figure 1B(a,b) the PLGA-DMC phase (stained with Nile Red) could still be sheared by the aqueous phase to form homogeneous droplets. After droplet formation, the sodium alginate solution would continue to carry the droplets out of the capillary and into the curing bath (calcium chloride solution) to form fibers. During this process, DMC in the droplet would continue to evaporate and PLGA would gradually cure to form microspheres. Due to the porous nature of the calcium alginate fibers, the volatilization of DMC would not be hindered by the hydrogel. From Figure 1B(c,d), it can be clearly observed that PLGA microspheres are encapsulated in the calcium alginate hydrogel to form the PLGA microsphere@calcium alginate fiber structure, and the microspheres show a spherical morphology with uniform size and spacing.

### 2.3. Effect of Flow Rate on Morphology of Microsphere Fibers

**Figure 2:**
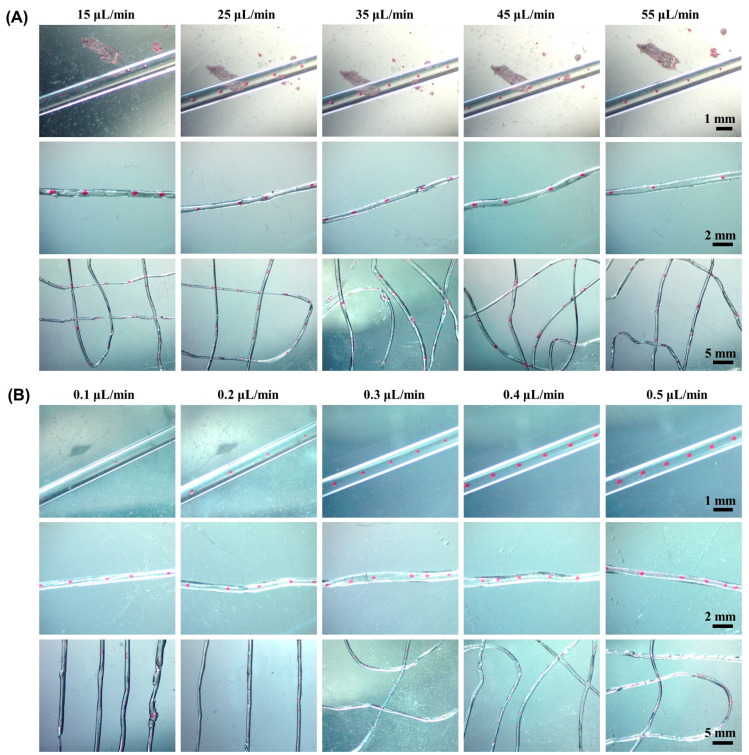
Droplet and microsphere alginate fibers at various flow rates of continuous phase (**A**) and dispersed phase (**B**).

The size and spacing of polymer microspheres in fibers can be controlled by changing the flow rate of the continuous phase and dispersed phase to meet different application requirements. In this section, the impact of both flow rates on fiber morphology was investigated. Firstly, the flow rate of the dispersed phase was fixed at 0.4 μL/min. When the flow rate of the continuous phase varied from 15 to 55 μL/min, it was observed that the size of the droplets and microspheres decreased and the spacing increased. Figure 2A shows the droplet (the first row) and the microsphere fibers (the second and third rows) under the conditions of different continuous phase flow rates. As the flow rate of the continuous phase increased, the shear force on the dispersed phase also increased, and the extrusion tendency of the dispersed phase was strengthened, so the size of droplets and microspheres decreased and the spacing increased.

Then a continuous phase flow rate was fixed at 50 μL/min, and the effect of dispersed phase flow rate varying from 0.1 to 0.5 μL/min on the size of droplets and microspheres was investigated. Figure 2B shows the droplet (the first row) and microsphere alginate fiber (the second and third rows) under different flow rates of the dispersed phase. It can be seen from the figure that with the increase of dispersed phase flow rate, the size of the droplet and microsphere increases and the spacing decreases. As the flow rate of the dispersed phase increased, the anti-shear effect was strengthened, so the droplet size increased, as well as the generation frequency, and the droplet spacing decreased.

**Figure 3:**
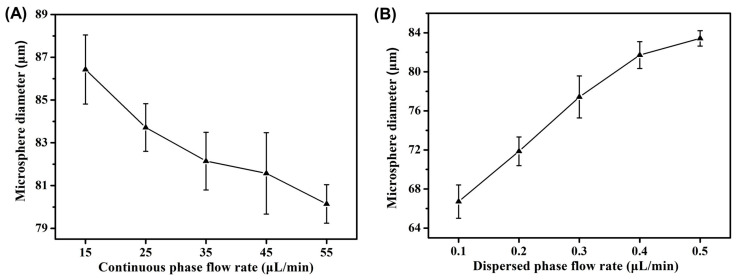
The influence of continuous phase flow rate (**A**) and dispersed phase flow rate (**B**) on the size of the microspheres.

The influence trend of the flow rate of the continuous phase and dispersed phase on the size of microspheres is shown in Figure 3A,B, respectively. In general, both flow rates have an impact on the size and spacing of droplets and microspheres. The flow rate can be adjusted according to the application requirements to prepare microspheres with different sizes and spacing. For example, the size of microspheres can be adjusted to achieve a series of drug loading and release kinetics, and the spacing of microspheres can be applied to fiber coding, etc. The manner of adjusting the size of microspheres by flow rate is a unique advantage of the microfluidic preparation method, which is simpler, faster and more accurate than conventional methods.

### 2.4. pH Responsive Properties of Microsphere Fibers

**Figure 4:**
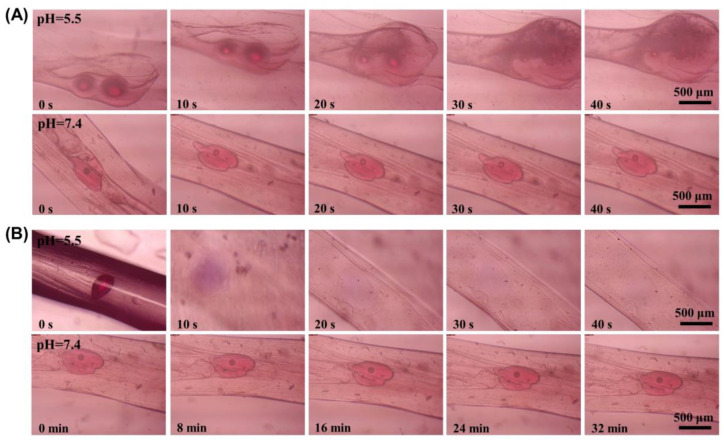
*p*(BMA-*co*-DAMA-*co*-MMA) microspheres@calcium alginate fibers (**A**) and *p*(BMA-*co*-DAMA-*co*-MMA) microspheres@CMC-doped calcium alginate fibers (**B**) under different pH values (pH = 5.5 and pH = 7.4).

As this work aims to construct a smart dressing with pH-responsive drug release properties, in order to impart pH-responsive properties to the microsphere fibers, a pH-sensitive polymer *p*(BMA-*co*-DAMA-*co*-MMA) was used as the microsphere matrix material, which is biocompatible and degradable under acidic conditions and relatively stable under neutral and alkaline conditions. This property makes the material particularly suitable for drug release in acidic environments, such as gastric and gynaecological drugs and for triggered drug release in acidic conditions such as tumorous and inflammatory tissues. The *p*(BMA-*co*-DAMA-*co*-MMA) microspheres@calcium alginate fibers were prepared by dissolving *p*(BMA-*co*-DAMA-*co*-MMA) in DMC to serve as the dispersed phase and Nile Red was added to indicate the location of the microspheres. As shown in Figure 4A, significant degradation of the microspheres in the fibers occurred within 40 s at pH = 5.5, while no significant degradation of the microspheres was observed at pH = 7.4. This result confirms that the *p*(BMA-*co*-DAMA-*co*-MMA) microspheres in the fiber can degrade and perform drug release in response to the change in environmental pH to acidic and that the hydrogel fiber shell does not affect the performance of the microspheres in response to environmental pH.

In addition, despite the inherent pH responsiveness of alginate hydrogels, the effect of carboxymethyl cellulose (CMC) doping on the pH-responsive properties of the fibers was investigated in order to enhance the pH sensitivity of the alginate fibers. CMC is well-known for its responsiveness to pH and ionic strength attributed to its polyelectrolyte nature and is a promising building block for bio-scaffolds, drug carriers and artificial tissues [28,29,30]. As shown in Figure S1, the swelling behavior of CMC-doped calcium alginate fibers in different pH environments was investigated. The fiber diameter increased with increasing pH when varied from 2 to 10 after 30 min of incubation. It indicates that fibers tend to shrink in acidic environments and swell in alkaline environments, a result that is consistent with literature reports [25,31]. The shrinking or swelling effect of fibers will have an impact on the release of internal drugs.

The degradation of microspheres in the CMC-doped microsphere fibers was then investigated at pH = 5.5 and pH = 7.4, respectively. As shown in Figure 4B, significant degradation of the microspheres in the fibers could occur in 20 s in the pH = 5.5 environment, while no obvious collapse of the microspheres was observed in the pH = 7.4 environment for 32 min. The diffusion of Nile Red released by *p*(BMA-*co*-DAMA-*co*-MMA) microspheres was faster in the CMC-containing fibers compared with the non-CMC-doped microsphere fibers. This result can be attributed to the fact that in an acidic environment, the crumpling of the CMC-containing fibers facilitated the diffusion of dye molecules released by the degradation of the microspheres to the exterior of the fibers. It is expected that as the acidity of the drug delivery site increases, the rate of drug release will be accelerated since the hydrogel shrinking will intensify.

**Figure 5:**
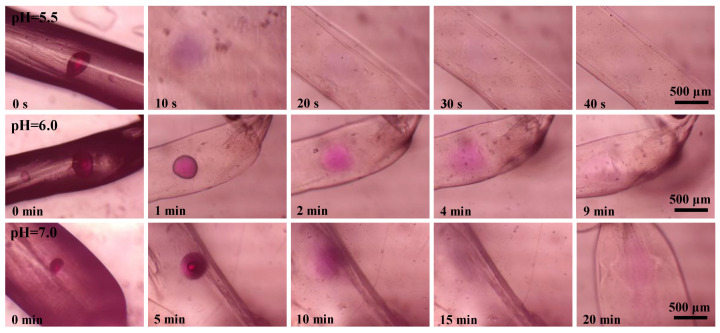
*p*(BMA-*co*-DAMA-*co*-MMA) microspheres@CMC-doped calcium alginate fibers at pH = 5.5, pH = 6.0 and pH = 7.0.

Drug carriers with pH-responsive properties have attracted considerable attention due to the fact that pH value differs in different tissues, such as the acidic condition of around pH 2 in the stomach, the weakly acidic tumor intracellular environment (pH∼5.5), and the basic condition in the small intestine with pH 7.2~7.5 [32,33]. Since the degradation rate of *p*(BMA-*co*-DAMA-*co*-MMA) microspheres and the degree of shrinking of the fibers differed under different pH conditions, the degradation of microspheres in CMC-doped fibers was compared under several physiological environmental pH conditions at pH = 5.5, pH = 6.0 and pH = 7.0. As shown in Figure 5, at pH = 5.5, the microspheres degraded significantly around 10 s and complete dye diffusion was observed around 20 s. At pH = 6.0, the microspheres degraded significantly around 2 min and complete dye diffusion was observed around 4 min, while at pH = 7.0, the microspheres degraded significantly around 10 min and complete dye diffusion was observed around 15 min. These results indicate that the degradation rate of the microspheres decreases and the dye diffusion time increases with increasing pH, which is a combination of the properties of the *p*(BMA-*co*-DAMA-*co*-MMA) polymer and the crumpling and swelling behavior of the CMC-doped fibers at different pH values. This property allows the microsphere fibers to have intelligent drug release rate modulation, for example, a decrease in environmental pH caused by conditions such as continued tumor cell proliferation or aggravating inflammation can trigger accelerated drug diffusion in the microsphere fibers, resulting in rapid inhibition of disease progression.

### 2.5. Controlled Drug Release Properties of Microsphere Fibers

**Figure 6:**
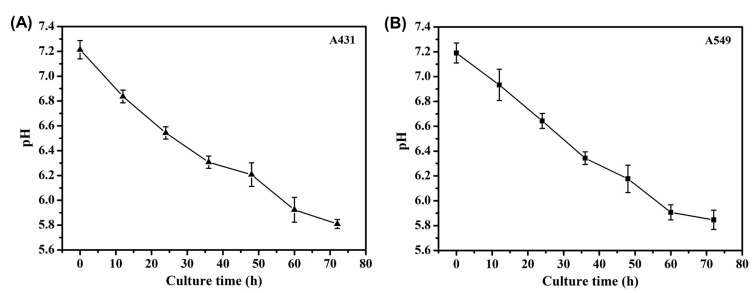
Change in pH of A431 cells (**A**) and A549 cells (**B**) with culture time.

In this work, the tumor cell environment was selected to test the triggered drug release performance of microsphere fibers in an acidic physiological environment. The pH of the culture environment of A431 cells (human skin squamous carcinoma cells) and A549 cells (human lung cancer cells) was first measured at regular intervals, and the results are shown in Figure 6. After 72 h of culture, the pH of the culture environment of A431 cells (Figure 6A) decreased approximately from 7.2 to 5.8, and that of A549 cells (Figure 6B) decreased approximately from 7.2 to 5.9. This result indicates that the tumor cell environment is acidic and it is presumed that microsphere fibers can undergo microsphere degradation and drug release in this environment.

**Figure 7:**
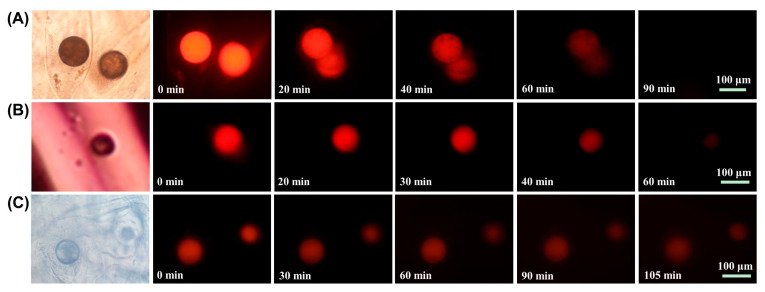
DOX release from microsphere fibers in the A431 (**A**) A549 (**B**) and normal skin cell (**C**).

Therefore, after 72 h of cell culture, microsphere fibers loaded with the chemotherapeutic drug doxorubicin (DOX) were added for co-culture, and the DOX release from the microspheres within the fibers was observed regularly using fluorescence microscopy. Figure 7A demonstrates the release of DOX from the microsphere fibers in the A431 cell environment. It can be observed that the fluorescence intensity of the microspheres gradually decreased with increasing incubation time, and the fluorescence of the microspheres almost disappeared at about 90 min of co-culture, indicating that DOX in the microspheres was gradually released and diffused. This result can be attributed to the degradation of the microspheres in the fibrils and the release of the drug in an acidic environment.

The DOX release of microspheres in the A549 cell environment was also tested, as shown in Figure 7B. During co-culture, the fluorescence of the microspheres gradually diminished and almost disappeared at approximately 60 min. This result suggests that microsphere fibers can also achieve triggered drug release in the lung tumor cell environment.

Finally, the co-culture of the microsphere fibers with normal skin cells was investigated, as shown in Figure 7C. At 105 min co-culture time, the microspheres still showed significant red fluorescence, indicating that no significant diffusion of DOX occurred within the microspheres. These results suggest that the drug-loaded microsphere fibers undergo rapid drug release in a tumor cell environment, while the drug release in a normal cell environment is low, allowing for environmentally selective drug release, a property that can protect normal tissue to some extent during therapeutic administration.

### 2.6. Discussion

In the preparation of microsphere alginate fibers, changes in flow rate mainly affected the size and spacing of the droplet and microsphere, while no significant effect on outer fiber diameter or morphology was observed. Compared with block or membrane-based dressings, fibrous materials have a larger specific surface area, which facilitates adequate contact of the dressing with the drug delivery site and drug release and the fibers are more easily placed in narrow spaces such as tubular tissues, or assembled to form more complex 3D bionic structures with multiple layers. The size of the microspheres should have a significant impact on degradation rate and drug release, which has not been explored in depth in this study due to the narrow variation range of microsphere size. This is due to the fact that although the flow rate has a remarkable impact on droplet size, the size of the microspheres decreases significantly compared with droplets after solvent volatilization, so the size of the microspheres changes less significantly than droplets with different flow rates.

In terms of the pH response of the microsphere fibers, this work also investigated the degradation and dye release of the microspheres at pH = 8, pH = 9 and pH = 10. The results showed that the microspheres were more stable in alkaline environments and the dye release was highly dependent on the slow erosion and dissolution of the carriers by the ambient aqueous solution. In addition, the loading rate of drug molecules in the microspheres is an important factor for drug diffusion. In this study, we only revealed a general drug diffusion pattern with a fixed loading rate, which can be flexibly adjusted for specific disease treatments. Furthermore, the hydrogel fiber shell is necessary, firstly, to immobilize the inner microspheres and prevent them from flowing to non-drug delivery sites. Secondly, to regulate the rate of drug release through the contraction or swelling of the gel network. Thirdly, to load water-soluble macromolecules such as proteins as co-treatment factors during the preparation process. The encapsulation of drug-loaded microspheres in the alginate fibers also eases the low loading efficiency of small molecules due to the pores of the hydrogel.

The method developed in this study allows for a “one-step” production of fibrous materials encapsulating drug-loaded microspheres, which is simpler and faster than the conventional method and provides greater control over the size and morphology of the microsphere fibers. Actually, a number of reports have successfully developed hydrogel structures with drug-loading particles inside, the majority of which are based on an additional process of drug-loading particle fabrication, by emulsion method, microfluidic method, electrospray method, etc. [34,35,36,37,38,39,40,41]. After dispersing the particles in hydrogel precursors, fibers or scaffolds would be generated by using methods of electrostatic spinning, 3D printing and molding, etc. Despite the advantages held by these methods, the approach proposed in this study may have a more straightforward and mild process. Additionally, the drug-loading spheres are evenly dispersed in the hydrogel matrix attributing to the highly controlled droplet formation in this study, which allows drug release in a more controllable manner.

Overall, this work has presented a proof-of-concept study on the development of pH-dependent drug delivery dressing, and some in-depth studies will be conducted in the following studies. For instance, the microsphere size and drug loading rate must be investigated in detail to clarify the drug delivery efficacy. Given the significant improvement in pH responsiveness of the alginate hydrogels by the introduction of CMC, the effect of CMC ratio on the pH sensitivity of the fibers, with a view to obtaining microsphere fibers with controllable drug release rate will be focused; an in vivo study is pivotal to reveal the potential of the proposed microsphere fiber in surgical dressing applications.

## 3. Conclusions

Alginate hydrogel fibers encapsulated with hydrophobic polymer microspheres were successfully prepared using a self-built coaxial capillary microfluidic system. The size and spacing of the microspheres can be flexibly adjusted to suit different applications by flow rate regulation. Based on the biocompatibility and pH-triggered degradation of *p*(BMA-*co*-DAMA-*co*-MMA) microspheres, the microspheres encapsulated in fibers could still degrade rapidly in an acidic environment at pH = 5.5, and the gel fiber shell did not affect the sensitivity of the microspheres to an acidic environment. The pH responsiveness of the fibers was enhanced by incorporating CMC into the sodium alginate gels. It can be attributed to the fact that the addition of CMC tightens the hydrogel fibers in an acidic environment and facilitates the diffusion of small molecules in a similar way to ‘squeezing’, and the lower the pH, the faster the release and diffusion of the loaded molecules from the microspheres. The rapid release of DOX from the microspheres in the tumor cell environment was observed after co-culture with DOX-loaded microsphere fibers, whereas no significant release of the drug from the microspheres was observed during co-culture of the microsphere fibers with normal skin cells. The drug-loaded microsphere fiber constructed in this study can be used as a therapeutic dressing for skin diseases, especially in acidic disease environments such as skin tumors and inflammations, where the fibers can automatically adjust the drug release rate in response to pH while protecting normal tissues from drug action to a certain extent.

## 4. Experimental Section

### 4.1. Construction of Coaxial Capillary Microfluidic Device and Preparation of Hydrogel Fibers

**Scheme 1:**
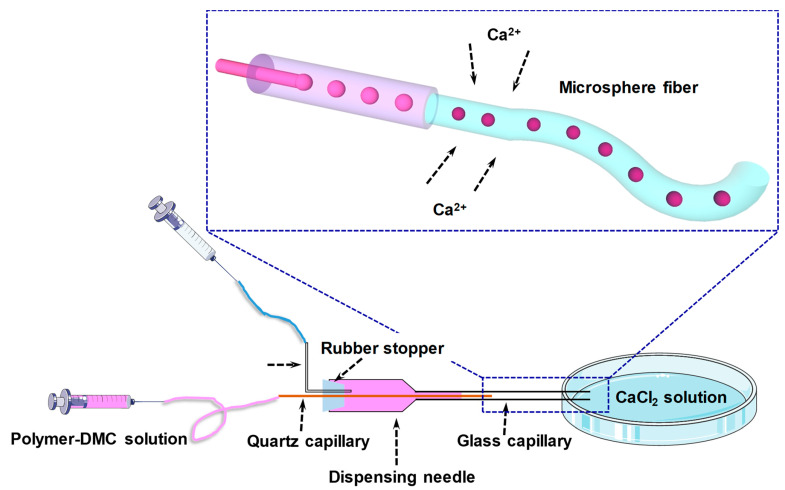
Schematic diagram of microfluidic preparation of the microsphere fiber.

The microfluidic device used in this study was constructed with a 20 G plastic dispensing needle, quartz capillary (i.d. 200 μm, o.d. 320 μm), glass capillary (i.d. 1 mm, o.d. 1.2 mm), silicone hoses and petri dish. Generally, a rubber stopper was used to seal the end of the dispensing needle, and two holes were punched in the rubber stopper. The quartz capillary was inserted in the central hole, and a syringe was connected to one end of the quartz capillary through a silicone hose to conduct the inner phase solution. The other end of the quartz capillary was coaxially fixed in the glass capillary through the tip of the dispensing needle. A stainless steel syringe was inserted into the other hole of the rubber plug and a syringe was connected to the outer end of the stainless steel syringe via a silicone hose to conduct the outer phase solution. Two micro syringe pumps were used for the transfer of the inner and outer phase solutions. The exit of the glass capillary was placed in a petri dish in which calcium chloride solution (10% wt) was added to create an outer layer of alginate fiber by ionic cross-linking. The device and formation process of the microsphere fiber is shown in Scheme 1.

### 4.2. Preparation of Dispersed Phase Solution

PLGA or *p*(BMA-*co*-DAMA-*co*-MMA) was mixed in DMC to a concentration of 1 mg/mL. The mixture was shaken for 20 min using an ultrasonic cleaner to fully dissolve the polymer, and then Nile Red was added to the solution at 0.1 mg/mL. For drug-laden composite fibers, doxorubicin hydrochloride (DOX) was added to the above *p*(BMA-*co*-DAMA-*co*-MMA) DMC solution at 1 mg/mL and 4% (*v*/*v*) triethylamine was added as a solubilizing agent and ultrasonically mixed.

### 4.3. Continuous Phase Solution Preparation

Sodium alginate was mixed in polyvinyl alcohol (PVA) solution (0.1% wt, serves as a surfactant to aid droplet formation) at 20 mg/mL and the mixture was stirred on a magnetic mixer for 10 h. For CMC-doped fibers, CMC was added to the aforementioned PVA solution at 15 mg/mL and stirred for 12 h.

### 4.4. Composite Fiber Preparation

Syringes, 5 mL and l mL, were used to load continuous phase solution and dispersed phase solution, respectively, and were placed on different microsyringe pumps with proper flow rate (for study on the impact of continuous phase flow rate, the dispersed phase flow rate was fixed at 0.4 μL/min, the continuous phase flow rate varied at 15, 25, 35, 45, 55 μL/min, respectively. For the study on the impact of dispersed phase flow rate, the continuous phase flow rate was fixed at 50 μL/min, the dispersed phase flow rate varied at 0.1, 0.2, 0.3, 0.4, 0.5 μL/min, respectively), and the preparation of fibers was observed in real-time through a stereomicroscope.

### 4.5. Microsphere Degradation at Different pH Conditions

PBS buffer solutions with pH = 2–10 were prepared, respectively, (2.85 g of Na_2_HPO_4_, 0.27 g of KH_2_PO_4_, 8.50 g of NaCl and 0.2 g of KCl were dissolved in 1 L deionized water to produce a pH 7.4 PBS buffer, and 2.0 mol/L NaOH or 2.0 mol/L KH_2_PO_4_ was used to adjust the pH to other values). The fibers loaded with *p*(BMA-*co*-DAMA-*co*-MMA) microspheres were incubated with each buffer, and the degradation of the microspheres and the diffusion of the dye were observed under a metalloscopic microscope at a regular time point.

### 4.6. Cell Culture

The surface of the T25 culture flask was first sterilized and then placed in a constant temperature cell culture incubator at 37 °C. When the cells were 90% fused, the T25 flasks were washed three times with PBS and digested by adding 0.5 mL of 0.25% trypsin. After digestion for 3 min, 1.5 mL of culture medium (high glucose DMEM cell base medium: Oricell standard grade fetal bovine serum = 9:1) was added to A431 cell (human skin squamous cell carcinoma cell) and skin cell culture flasks, and 1.5 mL of culture medium (1640 cell base medium: Oricell standard grade fetal bovine serum = 9:1) was added to A549 cell (human lung cancer cells) culture flasks, and 1:6 passages were performed. The A549 cell culture flasks were filled with 1.5 mL of culture medium (1640 cell basal medium: Oricell standard grade fetal bovine serum = 9:1) and passaged by 1:6.

### 4.7. Microsphere Degradation and Drug Release of Composite Fibers in Cell Environment

Skin cells, A431 cells, and A549 cells were cultured in 6-well plates. After the cells completely covered the bottom of the well plate, the composite fibers were placed into the corresponding wells (immersed in culture medium) for co-culture (1 mg fiber per mL culture medium), and the degradation of *p*(BMA-*co*-DAMA-*co*-MMA) microspheres and DOX diffusion in the fibers were observed regularly by fluorescence microscope.

## Data Availability

Data are contained within the article and Supplementary Materials.

## Supplementary Materials

The following supporting information can be downloaded at https://www.mdpi.com/article/10.3390/molecules29010193/s1. Materials, apparatus and Figure S1: Metallographic images of CMC doped calcium alginate fiber at various pH values.
