# Supplementary material for: Microfluidic Preparation of pH-Responsive Microsphere Fibers and Their Controlled Drug Release Properties
Source: Molecules. 2023 Dec 28;29(1):193. doi: 10.3390/molecules29010193 (PMC10780054; doi:10.3390/molecules29010193)
Supplement: Supplementary file 1 [file molecules-29-00193-s001.zip › molecules-2792698-supplementary.pdf]

Microfluidic Preparation of pH-responsive Microsphere Fibers and their Controlled Drug Release Properties

Ning Wang[1,2#], Yixuan Wei[3#], Yanrong Hu[4], Xiaoting Sun[1,2]*, Xiaohong Wang[1]*

[1] Center of 3D Printing & Organ Manufacturing, School of Intelligent Medicine, China Medical University, Shenyang 110122, China; wangning@cmu.edu.cn (N. W.)

[2] Department of Chemistry, School of Forensic Medicine, China Medical University, Shenyang 110122, China

[3] Teaching Center for Basic Medical Experiment, China Medical University, Shenyang 110122, China; weiyixuan07@126.com (Y.-X. W)

[4] Department of Biological Physics, School of Intelligent Medicine, China Medical University, Shenyang 110122, China; 18262637190@163.com (Y.-R. H.)

* Correspondence. xtsun@cmu.edu.cn (X.-T. S.); wangxiaohong@cmu.edu.cn or wangxiaohong709@163.com (X.-H. W.)

[#] Ning Wang and Yixuan Wei contributed equally to this work

Supporting Information Text

Table of contents:

1. Materials 1

2. Apparatus 2

3. Figure S1 3

Materials

Sodium alginate (low viscosity) was purchased from Sigma-Aldrich Corporation. Poly(butyl methacrylate-*co*-(2-dimethylaminoethyl) methacrylate-*co*-methyl methacrylate) (*p*(BMA-*co*-DAMA-*co*-MMA)) was purchased from Shanghai Dexiang Medicine Tech. Co., ltd. Poly(lactic-*co*-glycolic acid) (PLGA) (50:50, M_w 10,000) was purchased from Jinan Daigang Biomaterial Co., ltd., China , polyvinyl alcohol (PVA) and anhydrous ethanol were purchased from Aladdin Reagent Co. Ltd, China. Dimethyl carbonate (DMC) and Nile Red were obtained from Sinopharm Chemical Reagent Co. Ltd, China. Doxorubicin hydrochloride (DOX) and Carboxymethylcellulose V (CMC) were purchased from Shanghai Civic Chemical Technology Co., Ltd, China. Phosphate buffer saline solution (PBS, pH 7.4) was purchased from Beijing Solarbio Science Technology Co. Ltd, China. Human skin squamous carcinoma cells (A431) and normal human skin cells were obtained by the First Affiliated Hospital of China Medical University. Human pulmonary epithelial cells (A549) were obtained by School of Life Sciences, China Medical University. 18 MΩ cm water was used throughout.

Apparatus

Metallographic microscope (Shanghai YAN RUN Optical Machinery Technology Co., Ltd), Stereomicroscope was (New China Tengda Technology Co., Ltd), SW-CJ-IFD clean bench (Suzhou Purification Equipment Co.,Ltd). Micro syringe pumps (Baoding Lange Constant Flow Pump Co.,Ltd), Fluorescent microscopic images were taken on OLYMPUS TH4-200 fluorescent microscope (OLYMPUS, Japan).

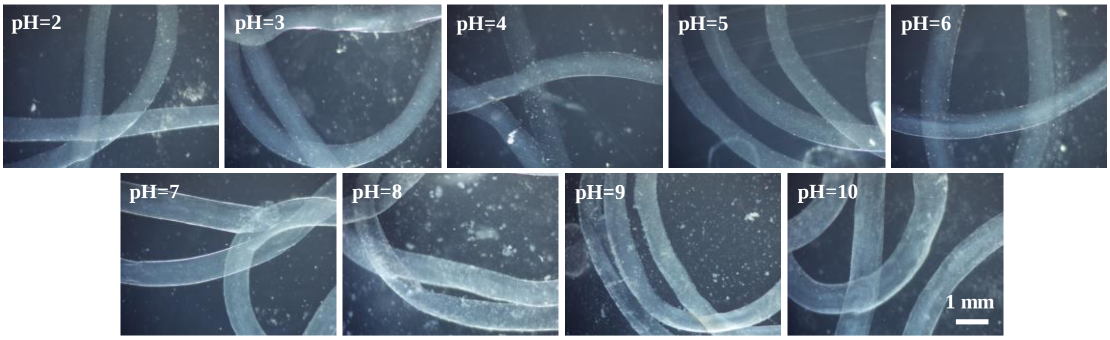

Figure S1. Metallographic images of CMC doped calcium alginate fiber at various pH values
